# Proteome Analysis of Daily Urine Samples of Pregnant Rats Unveils Developmental Processes of Fetus as Well as Physiological Changes in Mother Rats

**DOI:** 10.3390/biology14121700

**Published:** 2025-11-28

**Authors:** Haitong Wang, Linna Ge, Sijie Chen, Longqin Sun, Wei Sun, Youhe Gao

**Affiliations:** 1 Gene Engineering Drug and Biotechnology Beijing Key Laboratory, College of Life Sciences, Beijing Normal University, Beijing 100871, China; 2 Beijing Qinglian Biotech Co., Ltd., Beijing 100094, China

**Keywords:** urine, proteomics, pregnancy

## Abstract

Significant physiological changes occur in both the fetus and the mother during pregnancy. Urine proteins have been shown to reflect a wide range of physiological and pathological changes in the body. This study employed low-abundance protein-enriched magnetic nanobeads to conduct an in-depth analysis of the daily changes in urine proteins throughout the entire pregnancy of rats. Based on the 3455 identified urine proteins, fetal and maternal dynamic changes were observed in the pregnancy group compared to the control group, including blastocyst formation and cell division in the early stage of pregnancy. In addition, the morphogenesis- and development-related organs and tissues are significantly enriched at different stages of pregnancy. The 9th day after pregnancy is the stage when organ development is most concentrated, especially the nervous system and vasculature development. During the late stage of pregnancy, maternal-specific changes were observed, such as lactation. These results indicate that urine protein can reflect the fetal and maternal dynamic physiological alterations during pregnancy, which suggests the potential value of urine protein analysis in pregnancy health monitoring. It is emphasized that the analysis focuses on the daily variations in the urine proteins, as these daily changes are expected to reveal more dynamic and detailed information about the physiological processes during pregnancy.

## Simple Summary

Monitoring the dynamic changes in the mother and embryo during pregnancy is crucial for ensuring a smooth pregnancy. However, there are still difficulties in conducting early monitoring of these changes, and there are many limitations in directly conducting dynamic human pregnancy research. To address this issue, this study selected rats as experimental subjects. By analyzing the changes in urine proteome after pregnancy, we explored the dynamic changes in biological processes during pregnancy and embryo development. We collected urine samples from rats every day after pregnancy and used proteomics technology to compare the differences in proteins between pregnant rats and normal rats. Eventually, we found that some proteins showed significant changes along with the rat pregnancy process, and these changes could reflect fetal development and the physiological states of the mother adjusting to pregnancy, etc. This research not only provides a new basis for understanding the physiological changes in the mother during pregnancy but also offers important references for further exploring the mechanisms of pregnancy-related diseases in humans and developing indicators for pregnancy monitoring.

## 1. Introduction

Pregnancy is an extremely complex and precisely regulated physiological process, during which a series of significant changes occur in both the mother and the fetus. The maternal physiological systems need to make adaptive adjustments to meet the demands of fetal growth and development, while the fetus undergoes a gradual developmental process from a fertilized egg to a complete individual in the uterus [1,2]. The development before embryo implantation is the first step in nurturing a new life, including from the fertilized egg to the 2-cell, 4-cell, 8-cell, morula, and blastocyst stages [3]. The further development of the blastocyst requires establishing connections with the maternal uterine environment to obtain nutritional support. At this point, implantation of the blastocyst is necessary. This peri-implantation period is followed by the events of gastrulation, placentation, and postgastrulation organogenesis. This is a critical stage in embryonic development. During this period, the developmental blueprint (the body plan) is established through the ordered generation of various cell types and the assembly of precursor tissues that build body parts and organs [4]. At all stages of pregnancy, the occurrence of any abnormal situation may lead to serious consequences, jeopardizing the normal development of the fetus and the health of the mother. For example, placental dysfunction [5], maternal nutritional imbalance [6], or endocrine disorders [7] can lead to adverse outcomes, including premature birth, fetal growth retardation, and even miscarriage. Therefore, it is of great importance to monitor the pregnancy process as early, comprehensively, and meticulously as possible.

When the body is stimulated, urine is not controlled by the homeostatic regulation mechanism and can better retain the changes resulting from minor stimuli received by the body [8]. Currently, in clinical monitoring of fetal development, ultrasound examination is the primary method, which can provide information on various aspects of the fetal morphology and structure [9]. However, the monitoring effectiveness of ultrasound examination still has limitations, making it difficult to accurately capture earlier and more detailed embryonic development changes and potential abnormalities.

Urinary proteomics, as an emerging and highly promising research field, has attracted much attention due to its high sensitivity. Numerous studies have confirmed that the urinary proteome can effectively distinguish the occurrence and development of various diseases, including coronary artery disease [10], bladder cancer [11], glioma [12], autism [13], COVID-19 [14], etc. Urine has become an important source of biomarkers for disease diagnosis and monitoring, such as obesity-related metabolic diseases [15], chronic kidney disease [16], lupus nephritis [17], brain diseases [18], endometriosis [19], endometrial cancer [20], and prostate cancer [21]. And in the field of pregnancy, existing research has shown that information on embryonic development during pregnancy can be reflected in the urinary proteome [22]. However, the current research on the changes in the urinary proteome during the pregnancy of rats is still not detailed and comprehensive enough.

The aim of this study is to identify the day-by-day differences in the urinary proteome during the pregnancy of rats through comparative analysis so as to provide more abundant and accurate data support for a deeper understanding of the fetal and maternal physiological mechanisms of pregnancy.

## 2. Materials and Methods

### 2.1. Rat Caging

The 8-week-old Wistar rats (8 females and 4 males) were purchased from Beijing Vital River Laboratory Animal Technology Co., Ltd., (Beijing, China). All rats were housed in a standard environment (room temperature: 22 ± 1 °C, humidity: 65–70%). After acclimatization to the new environment for one week, the experiments commenced. Before that, none of the rats had ever mated. Male and female rats were caged at a ratio of 1:1 at 16:00 PM. The next day at 7:00 AM, the female rats were examined for vaginal plugs. The female rats with vaginal plugs were regarded as successfully mated.

All experimental operations complied with the review and approval of the Ethics Committee of the College of Life Sciences, Beijing Normal University, with the approval number CLS-AWEC-B-2022-003.

### 2.2. Urine Sample Collection

For the 8 female rats, urine was collected for the first time from 20:00 to 8:00 the day before the start of mating, which was recorded as the pre-pregnancy urine sample D0. Then 4 female rats were randomly selected as the pregnancy group, and the remaining 4 female rats as the control group. The female rats in the pregnancy group were caged with the male rats from 16:00 to 7:00 the next day, while the control group was not caged. From 20:00 to 8:00 on the same day, urine was collected for the second time from the 8 female rats. The pregnancy group was recorded as the first-day pregnancy urine sample E1, and the control group was recorded as the first-day non-pregnancy urine sample D1. Thereafter, urine was collected from the pregnancy group and the control group every day from 20:00 to 8:00 and recorded as E2—E18 and D2—E18, respectively. All the samples were immediately stored in a −80 °C freezer after collection.

### 2.3. Preparation of Urine Samples

Rat urine samples were processed with a Magicomics-AP-9 automated platform (Beijing Qinglian Biotech Co., Ltd., Beijing, China) using the Magicomics DMB assay kit (Beijing Qinglian Biotech Co., Ltd., China) following the manufacturer’s instructions. Briefly, 10 μL of DMB beads were washed with 150 μL of dilution solution, then 50 μL of urine sample and 100 μL of dilution solution were added. The mixture was incubated at 37 °C for 1 h with shaking at 1000 rpm. After incubation, the supernatant was removed by magnetic separation, and the DMB beads were washed three times with 150 μL of dilution solution. Subsequently, 50 µL of enzyme solution (containing Tris(2-carboxyethyl) phosphine (TCEP) and 2-Chloroacetamide (CAA)) was added to resuspend the DMB beads, followed by digestion with 0.5 µg of trypsin at 37 °C for 4 h. After incubation, the mixture was placed on a magnetic separator to settle, and 45 µL of supernatant was transferred to a new centrifuge tube. Then, 25 µL of loading solution and 25 µL of stop solution were added and mixed. The entire liquid was loaded onto a desalting column, and washed with 100 µL of wash solution 1 and 100 µL of wash solution 2. After changing the centrifuge tube, 100 µL of elution solution was added, and the eluate was collected, freeze-dried, and analyzed using LC-MS/MS.

### 2.4. Proteome Analysis of Urine Samples

LC-MS/MS analysis was conducted using a timsTOF HT mass spectrometer (Bruker, Bremen, Germany) coupled with an UltiMate 3000 liquid chromatography system (Thermo Fisher Scientific, Germering, Germany). For each sample, 400 ng of peptides dissolved in 0.1% formic acid (FA) aqueous solution was injected. The liquid chromatography conditions included a C18 reverse-phase analytical column (C18, 1.5 μm, 100 μm × 15 cm). Mobile phase A consisted of 0.1% FA, while mobile phase B comprised 80% acetonitrile and 0.1% FA. The gradient was as follows: 0–17 min (3.5–32% B), 17–18 min (32–95% B), 18–20 min (95% B), and 21–22 min (95–1% B).

All the samples were analyzed in data-independent acquisition (DIA) mode with a mass scan range of *m*/*z* 300–1500 and a primary mass resolution of 60,000 (at 1222 *m*/*z*). In the TIMS tunnel, an accumulation time of 50 ms was set. The capillary voltage was adjusted to 1.5 kV, and the ion mobility ranged from 0.70 to 1.30 cm^2^/(V). The total cycle time was 1.23 s. For library construction, 6 fractions of pooled samples were analyzed in Data-dependent acquisition (DDA) mode with a mass scan range of *m*/*z* 100–1700, an accumulation time of 100 ms in the TIMS tunnel, a capillary voltage of 1.6 kV, an ion mobility ranging from 0.6 to 1.6 cm^2^/(V), and a total cycle time of 1.1 s with 10 PASEF cycles.

The raw data were analyzed with Spectronaut software (version 18.1.230626.50606) using the UniProtKB Rat database (1 March 2023 released) and a hybrid library constructed by the fractionated pooled samples. The parameters were set as follows: the protease was specified as trypsin with 2 missed cleavage sites allowed; variable modifications included methionine oxidation (Oxidation (M)) and protein N-terminal acetylation (Acetyl (Protein N-term)); the fixed modification was cysteine carbamidomethylation (Carbamidomethyl (C)); the peptide false discovery rate (FDR) was less than 1%. Data filtering was set to a Q-value of 0.01, and normalization was performed using local normalization.

### 2.5. Data Processing and Bioinformatic Analysis

After removing values less than 1, the quantitative data of all the samples were normalized using the median value of common proteins, which were identified in all samples [23]. Proteins detected in all the pregnant rats were retained for further analysis for at least one day. Missing values were imputed with a global minimum.

All the data were log2 transformed and analyzed by the R package Limma (version 3.58.1) for differential expression analysis between the pregnant group and the normal group across all time points from day 1 to day 18. The thresholds for differential proteins were set as follows: *p* < 0.05, |fold change| ≥ 1.5, and no difference between the two groups at day 0. Enrichment analysis of biological processes for differential proteins at each time point was performed using the hypergeometric distribution [23]. The filtering criteria for enriched terms were *p* < 0.05, background term number ≥ 3, foreground term number (count) ≥ 2, and ratio > 0.1. The enrichment analysis of the entire gestation period (days 1–18) highlighted only the top 10 terms with the most significant *p*-values.

## 3. Results and Discussion

**Figure 1:**
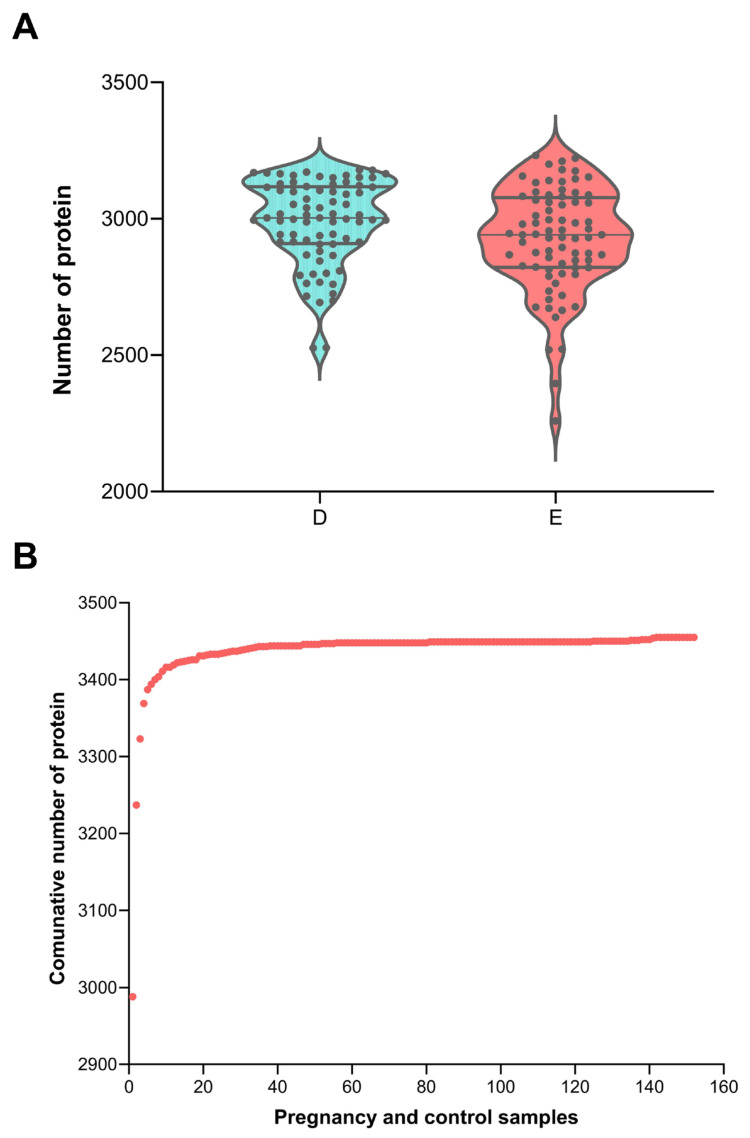
Urine protein identification result. (**A**) Number of identified urine proteins in each sample (D: control group, E: pregnancy group). (**B**) Accumulation curves of proteins identified in all the samples. A dot represents a sample.

In this study, a total of 152 urine samples were collected from rats in the gestation group (*n* = 4) and the control group (*n* = 4) before (day 0) or during the gestation period (days 1–18). After low-abundance protein enrichment by DMB beads, 3455 proteins were identified in all samples (Table 1 and Table S1, Figure 1A), with an average of 2956 proteins per sample. Compared with other studies using multiple fractionation [24] or extended MS gradient (e.g., 120 min) [25], our study achieved the highest number of rat urine protein identifications in only a 22 min MS run.

### 3.1. The Changes in Urine Protein in Pregnancy Rats and Control Rats

**Figure 2:**
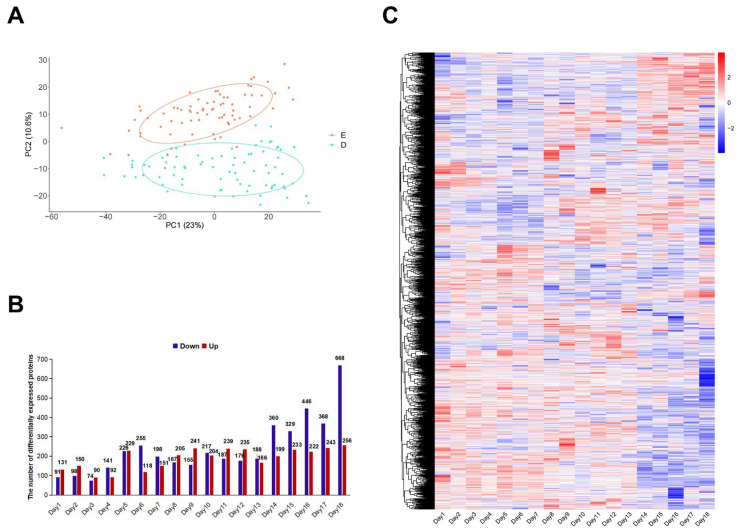
Difference of protein expression between the pregnancy group and the control group. (**A**) PCA analysis of all the samples (D: control group, E: pregnancy group). (**B**) Differentially expressed proteins (DEPs) between the pregnancy group and the control group at 1–18 days. (**C**) Heatmap of DEPs between the pregnancy group and the control group from days 1–18 after fertilization.

After data filtering, 3201 proteins were retained in the subsequent analysis. The PCA result suggested the difference of urine protein expression between the pregnancy group and the control group (Figure 2A). Further analysis discovered differentially expressed proteins (DEPs) in urine samples between the two groups from days 1–18 (Figure 2B, Table S2). The number of DEPs showed three peaks: 1–2 days after fertilization, 5–7 days after fertilization (embryo implantation), and 14–18 days after fertilization (before delivery), indicating that there were significant changes in urine proteins during the gestation period, particularly at these three stages. The heatmap also showed the expression changes in all the DEPs from days 1 to 18 after fertilization (Figure 2C).

### 3.2. The Changes Occurring Throughout the Entire Gestation Period in Rats

**Figure 3:**
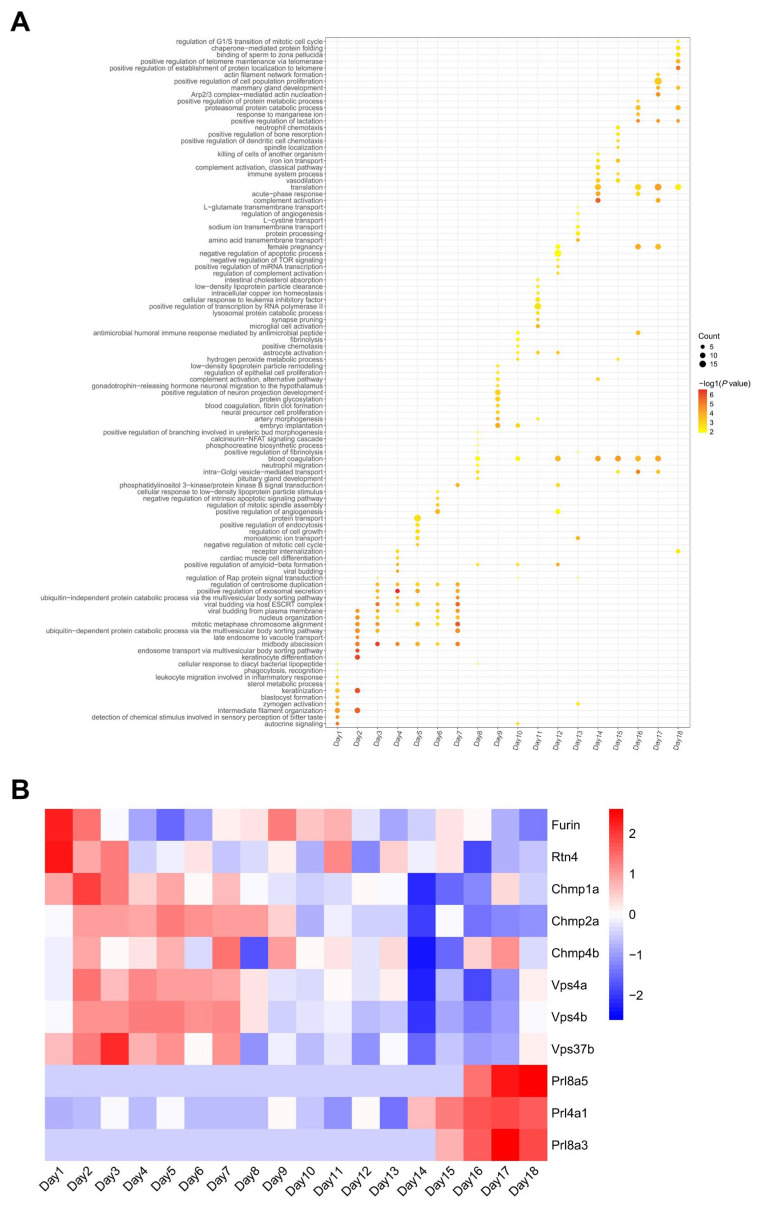
Biological functions (**A**) and related DEPs (**B**) of up-regulated rat urinary proteins during the gestation.

GO-BP enrichment analysis of DEPs from days 1–18 showed that, compared with control rats, the urinary proteome of pregnant rats gradually exhibited regularly up-regulated biological functions during certain stages of pregnancy (Figure 3, Table S3). Corresponding to blastocyst formation and implantation into the uterus after fertilization in early gestation, blastocyst formation was enriched as early as the first day after fertilization. Biological processes associated with cell division were significantly enriched from days 2 to 7, including midbody abscission, mitotic metaphase chromosome alignment, and regulation of centrosome duplication. Additionally, polysomal sorting-related processes were also enriched during this period, such as positive regulation of exosomal secretion, ubiquitin-independent protein catabolic process via the multivesicular body sorting pathway, and ubiquitin-dependent protein catabolic process via the multivesicular body sorting pathway. Previous studies have demonstrated that MVBs can specifically recognize and mediate the degradation of sperm mitochondria-derived materials (MD) [26].

In the intermediate and mid-to-late phases of the pregnancy period following embryo implantation, biological processes related to embryo development continuously appeared in the top 10 enriched terms. For example, pituitary gland development and positive regulation of branching involved in ureteric bud morphogenesis were observed on day 8. Artery morphogenesis, neural precursor cell proliferation, positive regulation of neuron projection development, and gonadotrophin-releasing hormone neuronal migration to the hypothalamus were enriched on day 9 of pregnancy. These results may reflect the embryonic development of the renal system, vascular system, and nervous system during pregnancy [27,28,29].

Additionally, from the middle of gestation, especially in the late stages of pregnancy, blood coagulation remained active in the pregnancy group. This result is consistent with gradually increased coagulation function from early to late pregnancy [22], which prevents excessive bleeding during delivery. Fibrinolysis increased around day 10, which is consistent with elevated antithrombin time during and after fetal organogenesis to prevent thrombus formation [30]. Concurrently, we observed enrichment of terms related to complement activation during this phase, including complement activation alternative pathway, complement activation-classical pathway, and regulation of complement activation. Complement activation exerts multifaceted functional roles during pregnancy, including protective and destructive actions at the placental level, complement activation at the fetal–maternal interface to defend against pathogens, and aiding in the removal of apoptotic and necrotic cells [31].

At the end of gestation, the stage before delivery, the biological processes associated with lactation were enriched on days 16–18. The mammary gland is a dynamic organ regulated by reproductive and metabolic hormones, developing from puberty and forming a branched, milk-secreting structure at the end of pregnancy. Lactation begins post-placental delivery with progesterone withdrawal, is sustained by increased prolactin and oxytocin secretion, and is stimulated by infant suckling [32,33]. This represents a specific change during pregnancy that has not been noted in previous studies of urine proteins in pregnant rats [22].

Changes in key proteins in the biological processes described above were shown in Figure 3B, including proteins Furin and Rtn4 associated with blastocyst formation [34,35]; Chmp family proteins (Chmp1a, Chmp2a, and Chmp4b) and Vps family proteins (Vps37b, Vps4a, and Vps4b) associated with cell division [36]; and Prl family proteins (Prl4a1, Prl8a3, and Prl8a5) associated with lactation [37].

### 3.3. Dynamic Changes in Embryonic Development Throughout the Whole Pregnancy

#### 3.3.1. Embryonic Development

**Figure 4:**
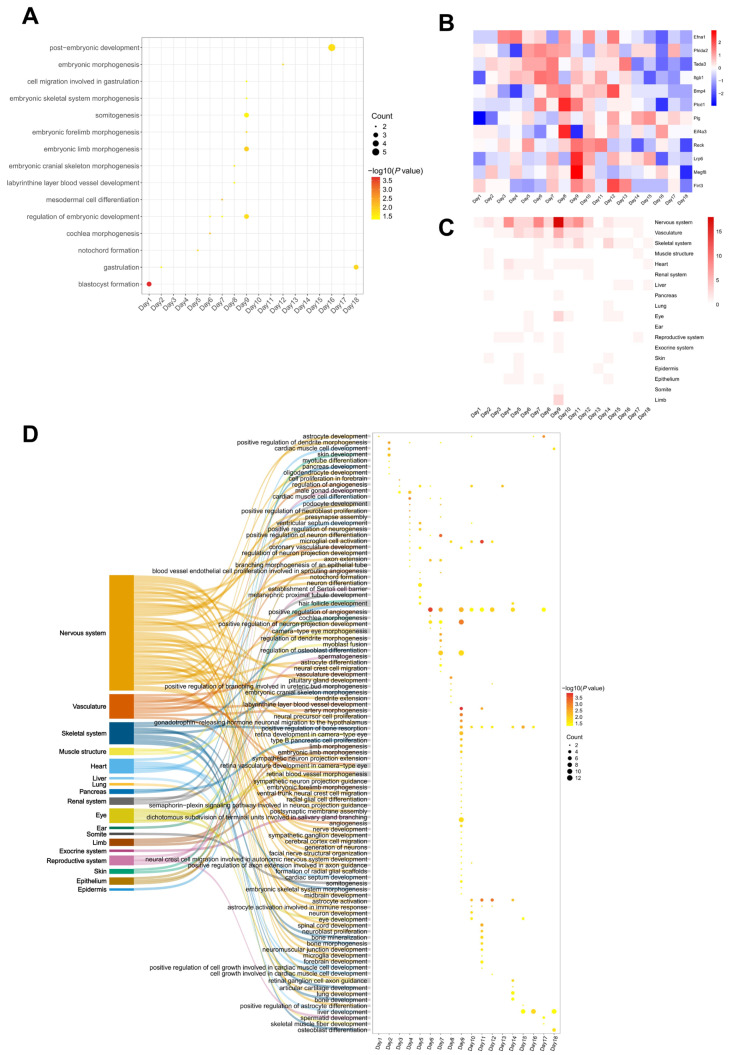
Dynamic changes in enriched biological processes related to embryonic development in pregnant rat urine proteins. (**A**) Biological processes directly related to embryonic development. (**B**) Heatmap of proteins related to embryonic development. (**C**) Number of biological process terms related to organs were aligned to the development of 18 organs during the pregnancy period. (**D**) Specific biological processes related to the development of 18 organs, different colors correspond to different organs.

Since biological processes related to embryo development were continuously enriched in the top 10 terms during pregnancy, we further investigated all enriched terms related to embryonic development throughout days 1–18 of gestation. In addition to blastocyst formation observed on day 1, other biological processes directly linked to embryonic development were also discernible in the rat urinary proteome (Figure 4A, Table S4). For example, events related to gastrula and placenta development, including gastrulation, mesodermal cell differentiation [38], cell migration involved in gastrulation [39], and labyrinthine layer blood vessel development [40,41,42]. Other events related to morphogenesis of anatomical structure were also observed during pregnancy, including notochord formation [43], cochlea morphogenesis [44,45,46], embryonic cranial skeleton morphogenesis [47], embryonic limb morphogenesis and embryonic forelimb morphogenesis [48,49], embryonic skeletal system morphogenesis [50], and embryonic morphogenesis. At the late developmental stage, the post-embryonic development term occurred on day 16, whose specific outcome is the completion of embryonic development to the mature structure.

Changes in key proteins in the biological processes described above were shown in Figure 4B, including Efna1 associated with notochord formation [51]; Phlda2 and Tada3 associated with the regulation of embryonic development [52,53], Itgb1 and Bmp4 associated with mesodermal cell differentiation [54,55,56]; Plcd1 and Plg associated with the labyrinthine layer blood vessel development [57,58]; Bmp4 and Eif4a3 associated with embryonic cranial skeleton morphogenesis [59,60]; Lrp6, Reck, and Megf8 associated with embryonic limb morphogenesis [61,62,63]; and Flrt3 and Bmp4 associated with embryonic morphogenesis [64,65].

#### 3.3.2. Organ Development of Embryo

To explore the developmental status of embryonic organs and tissues throughout gestation, we systematically summarized terms associated with organ and tissue development from days 1 to 18, utilizing keywords pertinent to tissue and organ development, such as organ development, cell differentiation of organ, organ formation, organ morphogenesis. Throughout the entire gestation, we summarized enriched biological processes into the development of 18 distinct embryonic organs and tissues (Figure 4C,D), including tissues in the nervous system, vascular system, and skeletal system, as well as principal organs such as the heart and liver. Surprisingly, terms related to the nervous system and vascular system were almost present throughout the entire gestation period of rats, particularly those related to the nervous system [66,67]. Terms related to the development of the heart [68,69,70] and kidney system [71,72] were significantly enriched in the early and middle stages of gestation, while those related to liver development [73,74] were more pronounced in the later stage. Terms related to somitogenesis [75,76] and limb development [48,49] were enriched on day 9 of pregnancy. These observations in the urine of pregnant rats indicate that dynamic changes in embryonic development are directly reflected in the alterations of the maternal urinary proteome. These results also provide a new perspective for monitoring human pregnancies. At the same time, this research has some limitations; the sample size for each group in this study was *n* = 4, which is relatively small in terms of statistical power. And the findings in rats need to be further verified in human beings.

## 4. Conclusions

In this study, daily urinary proteome alterations during rat pregnancy were analyzed. Compared with the control group, changes associated with blastocyst formation and cell division were observed in the early stage of pregnancy. In the intermediate and mid-to-late phases of the pregnancy, changes associated with embryonic development and organ morphogenesis were revealed. Maternal-specific changes, such as lactation, were found in the late stage of pregnancy. These results suggest that both fetal and maternal physiological alterations during pregnancy can be detected in the urinary proteome.

In conclusion, based on the data of rat urine proteomics, we decoded the dynamic correlation between rat pregnancy and embryonic development. During rat pregnancy, the significant physiological changes that occur in the embryo and the mother can both be detected through urine protein profiles. Our findings provide important clues for tracking the rat embryonic organ development and potential early detection of pregnancy abnormalities, which provide a foundation for future research of urine proteomics in the human pregnancy process.

## Figures and Tables

**Table 1:** Urine protein identification result.

Group	Number of Samples	Time Point	Number of Identified Proteins
Pregnant group	*n* = 76	Day 0–Day 18	2924 ± 192
Control group	*n* = 76	Day 0–Day 18	2987 ± 155
All	*n* = 152	Day 0–Day 18	2956 ± 177

## Data Availability

The datasets in this study will be available upon acceptance for formal publication (ID: IPX0011105002, https://www.iprox.org/).

## Supplementary Materials

The following supporting information can be downloaded at https://www.mdpi.com/article/10.3390/biology14121700/s1: Table S1: Quantitative results of rat urine proteins; Table S2: Results of differentially expressed urine proteins between pregnant group and control group; Table S3: Biological function enrichment results (top 10 terms) of differentially expressed proteins; Table S4: Biological function enrichment results of differentially expressed proteins with terms directly related to embryonic development.
